# Developing a competency-based curriculum in HIV for nursing schools in Haiti

**DOI:** 10.1186/1478-4491-6-17

**Published:** 2008-08-29

**Authors:** Elisa Knebel, Nancy Puttkammer, Adrien Demes, Ruth Devirois, Mona Prismy

**Affiliations:** 1 International Training and Education Center on HIV (I-TECH), University of Washington, 901 Boren Avenue, Suite 1100 Seattle, WA 98104-3508, USA; 2 International Training and Education Center on HIV (I-TECH)/Haiti, Delmas 95, Route de Jacquet #14, Petion Ville, Haiti; 3 Institut Haïtien de Santé Communautaire, Angle rues Rigaud et Lambert, Pétionville, B.P. 13408, Haiti

## Abstract

**Background:**

Preparing health workers to confront the HIV/AIDS epidemic is an urgent challenge in Haiti, where the HIV prevalence rate is 2.2% and approximately 10 100 people are taking antiretroviral treatment. There is a critical shortage of doctors in Haiti, leaving nurses as the primary care providers for much of the population. Haiti's approximately 1000 nurses play a leading role in HIV/AIDS prevention, care and treatment. However, nurses do not receive sufficient training at the pre-service level to carry out this important work.

**Methods:**

To address this issue, the Ministry of Health and Population collaborated with the International Training and Education Center on HIV over a period of 12 months to create a competency-based HIV/AIDS curriculum to be integrated into the 4-year baccalaureate programme of the four national schools of nursing.

**Results:**

Using a review of the international health and education literature on HIV/AIDS competencies and various models of curriculum development, a Haiti-based curriculum committee developed expected HIV/AIDS competencies for graduating nurses and then drafted related learning objectives. The committee then mapped these learning objectives to current courses in the nursing curriculum and created an 'HIV/AIDS Teaching Guide' for faculty on how to integrate and achieve these objectives within their current courses. The curriculum committee also created an 'HIV/AIDS Reference Manual' that detailed the relevant HIV/AIDS content that should be taught for each course.

**Conclusion:**

All nursing students will now need to demonstrate competency in HIV/AIDS-related knowledge, skills and attitudes during periodic assessment with direct observation of the student performing authentic tasks. Faculty will have the responsibility of developing exercises to address the required objectives and creating assessment tools to demonstrate that their graduates have met the objectives. This activity brought different administrators, nurse leaders and faculty from four geographically dispersed nursing schools to collaborate on a shared goal using a process that could be easily replicated to integrate any new topic in a resource-constrained pre-service institution. It is hoped that this experience provided stakeholders with the experience, skills and motivation to strengthen other domains of the pre-service nursing curriculum, improve the synchronization of didactic and practical training and develop standardized, competency-based examinations for nursing licensure in Haiti.

## Background

In light of severe physician shortages in the developing world, the World Health Organization's strategic framework for the emergency scale up of antiretroviral therapy (ART) involves training a range of health-care staff to support the delivery and monitoring of HIV/AIDS treatment. 'Task shifting' is the name given to a process of delegation whereby tasks are moved, where appropriate, to less specialized health workers [1].

Task shifting has lead nurses to be heavily involved in performing HIV testing and counselling, assessing patients for ART eligibility, assessing toxicity and treatment failure, and providing patient education, psychosocial support and adherence support [2]. Nurses may also play a lead role in record keeping and reporting. As the volume of patients under HIV/AIDS care and treatment services grows and services are decentralized, nurses may experience a shift of responsibilities, with even larger roles in initial evaluation and staging of patients, ART initiation, and patient monitoring [3].

As nurses are becoming increasingly central points of contact for clinical care of people living with HIV and AIDS (PLWHA), they must first be ensured adequate preparatory education. Scattered reports have shown, however, that most nurses in developing countries are not well prepared during their pre-service education in the knowledge, skills and attitudes needed to provide quality HIV/AIDS-related care [4,5].

Preparing nurses to confront the HIV/AIDS epidemic is a need in Haiti where the HIV prevalence rate is 2.2% [6], approximately 10 100 patients are currently receiving antiretroviral treatment [7], and there is a critical shortage of doctors, leaving nurses as the primary care providers for much of the population.

Haiti has four national nursing schools, graduating approximately 120 registered nurses per year. These schools face under-resourced infrastructure (few textbooks and teaching materials and little classroom space), variable quality of teaching with few classroom instructors prepared to educate, and few clinical instructors and sites available for clinical skills practice. Graduates often must do much of their learning on-the-job during their rotations, under limited supervision. Specific to HIV/AIDS education, a recent assessment revealed that related content is very loosely woven throughout the courses, and that inclusion of HIV is arbitrarily dependent on the interest of the faculty member assigned to the course, with key areas, such as HIV/AIDS counseling, prevention of mother-to-child transmission, and ART adherence, being largely overlooked [8].

Since 2004, the International Training and Education Center on HIV (I-TECH) has worked in Haiti to build capacity to respond to the AIDS epidemic. I-TECH is a collaboration between the University of Washington and University of California San Francisco and was established by the Health Resources and Services Administration (HRSA) in collaboration with the Centers for Disease Control and Prevention (CDC).

In June 2006, the Haitian Ministry of Health and Population (MSPP), specifically the directorate that is in charge of health science education, the *Direction de Formation et de Perfectionnement en Sciences de la Santé *(DFPSS), and I-TECH started a process of integrating current HIV/AIDS knowledge, skills and attitudes into the current curriculum using a competency-based approach. This article details the steps undertaken to develop, integrate and implement the new curriculum.

## Methods

In June 2006, DFPSS and I-TECH convened deans of the four public nursing schools, officials from the Haiti ministries of health and education, and selected education and HIV/AIDS experts to reflect on the status of HIV/AIDS-related education at the nursing schools and how to quickly address new content into an already overloaded curriculum in a resource-strained environment.

The stakeholders chose to form two committees – a coordinating committee made up of school heads and ministry officials that would ensure broad-based support and integration of the new topic into the existing curriculum and an eight-member curriculum committee made up of Haitian nurse educators, nurse trainers and one nurse HIV/AIDS expert to draft the new curriculum.

Upon review of other international projects and the education literature on various models of curriculum development and integration, stakeholders opted to use a competency-based approach for the integration process.

A competency is defined as the blend of skills, abilities, and knowledge needed to perform a specific task [9]. In both developed and developing countries, the traditional approach to nursing pre-service education has been for teachers to determine what content needs to be learned, teaching it, and then testing to see if the content was learned. This approach, though long established, does not guarantee that teachers use content reflecting the needs of the workplace and often relies on passive memorization from lectures as the dominant learning method for students. The literature is full of calls for curriculum reform in nursing education, advocating curricula that are responsive to changes in the health care delivery system, are research-based, are collaborative, and apply pedagogical innovation [10].

Recent reforms support the application of competency-based education – defining, teaching, and assessing competencies and then assessing student performance in relation to these, thus focusing on the outcome of the education, rather than on the process of the education (applying knowledge and skills rather than merely gaining knowledge) [11]. Experience shows that using competencies to define what is taught in the pre-service arena can achieve the following: provide clarity of learning direction for both faculty and students, set the framework for assessment, enable the curriculum to reflect the "real world" skills required to meet the health needs of the population and clarify the role of nurses vis-à-vis the other health professions [11-15].

**Figure 1:**
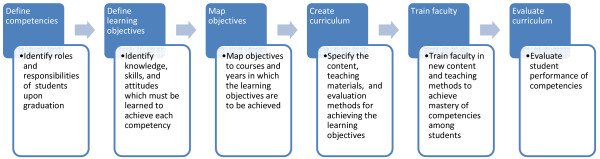
Competency-based education model.

A competency-based education model starts by asking the question: What will the nurse do on the job? Once this is known, specifications of learning objectives for instruction are derived. If integrating a new theme into an existing curriculum, these learning objectives can then be mapped to existing courses. Then, appropriate teaching and assessment methods are derived that will ensure mastery of the objectives, and faculty are trained in and oriented to the new curriculum. Finally, evaluation is conducted to ensure that students achieve mastery of the competencies. A schematic representation of this model appears in Figure 1.

## Results

When the curriculum committee began to design the new curriculum, the initial hurdle was to articulate and reach consensus on the HIV/AIDS competencies relevant for nurses. In-depth discussions among the participating experts at the beginning of the project, who had a good overview of the ongoing HIV/AIDS activities in Haiti, helped to identify a draft list of general competencies. Then, the curriculum committee reviewed HIV competencies relevant for developing country settings, which were drafted by the World Health Organization [16] and the National HIV Nursing Association [17] in the United Kingdom. Over a series of seven meetings, the committee, through facilitated discussions, adapted these competencies to the Haitian environment and formed a final list of five main HIV/AIDS competencies and 35 associated sub-competencies as shown in Table 1.

**Table 1:** HIV Competencies and sub-competencies

Competencies	Sub competencies
A. Prevent HIV infection among individuals and the community	A.1 Conduct community and individual education on HIV/AIDS
	A.2 Perform HIV pre-test counseling
	A.3 Conduct HIV testing
	A.4 Perform HIV post-test counseling
	A.5 Prevent and treat accidental blood exposure
	A.6 Prevent Mother-To-Child-Transmission of HIV
	A.7 Prevent and treat sexually transmitted diseases
	A.8 Ensure post-exposure prophylaxis in cases of sexual violence

B. Promote the health of people living with HIV	B.1 Provide counseling on well-being and nutrition
	B.2 Prevent opportunistic infections

C. Evaluate the health status of people living with HIV	C.1 Identify the clinical signs of HIV infection
	C.2 Conduct biologic tests
	C.3 Classify the patient according to stages of infection as defined by WHO and the CDC

D. Ensure the care of adults and children infected with HIV/AIDS	*D.1 Therapeutic interventions*
	D.1.1 Identify the patients eligible for ART
	D.1.2 Counsel for adherence to ART
	D.1.3 Treat opportunistic infections
	D.1.4 Manage the nutrition of PLWHA
	D.1.5 Administer ART
	D.1.6 Ensure the follow-up of a patents taking ART
	D.1.7 Manage a pregnant women infected by HIV
	D.1.8 Manage a child infected by HIV
	D.1.9 Provide palliative care
	
	*D.2 Psychosocial and community interventions*
	D. 2.1 Provide spiritual support
	D. 2.2 Provide social and economic support
	D. 2.3 Provide psychological support
	D.2.4 Support clients in managing grief
	D. 2.5 Link patients to legal support
	D. 2.6 Provide support to orphans and other vulnerable children
	D. 2.7 Ensure the community management of people living with HIV
	D.2.8 Prevent and treat burn-out among n

E. Ensure the management of various aspects of the HIV/AIDS control program	E.1. Work as a member of a multidisciplinary team
	E.2. Manage the drugs and other inputs necessary for the care of people living with HIV
	E.3. Manage the data entry of HIV patients
	E.4. Utilize the resources provided in the national AIDS control program
	E.5. Evaluate the activities of the national AIDS control program

The committee then defined the associated learning objectives for each sub-competency. Over 350 learning objectives were defined, with each sub-competency having multiple knowledge, skill and attitudinal learning objectives. The committee then mapped each of these objectives to existing courses in the overall nursing curriculum. Hours were not taken from existing courses to specifically make room for the new objectives, nor were additional hours added to the curriculum, but rather, the new HIV/AIDS-related learning objectives were integrated into the current courses. For example, in the Counseling and Communication course, when discussing how to use job aids and demonstrate good interpersonal skills, the faculty member is prompted to use an example of ART adherence counseling.

The learning objectives build upon each other at different stages in the curriculum. For example, a student nurse in year 1 will describe the relationship between HIV and nutrition, but by the years 3 and 4, she or he is able to define dietary needs of specific sub-groups of PLWHA and how to educate patients on specific meal preparation.

To support faculty with up-to-date HIV/AIDS content, the committee drafted an 'HIV/AIDS Reference Manual' that features evidence-based national and international core content, protocols, and guidelines for faculty to access.

The committee also drafted a 'HIV/AIDS Teaching Guide' organized by curriculum year and course, which was approved for dissemination to the nursing schools by the MSPP in November, 2007. For each course, the associated competency and sub-competencies, learning objectives, recommended chapters of the 'Reference Manual' or other materials, learning methods and evaluation methods are listed. An excerpt of a plan for a specific course is shown in Table 2.

**Table 2:** Sample course plan: infectious diseases, year 2

Competency	Sub-competency	Learning objectives	Content source	Learning methods	Assessment method
A. Prevent HIV infection among individuals and the community	A.5 Prevent and treat accidental blood exposure (ABE)	Describe the role of nurse in the prevention and treatment of ABE (K)	Chapter 3 in HIV Reference Manual	Large group discussion and lecture	Written exam
	
		Indicate the risks and degree of risk of ABE (K)	Chapter 3 in HIV Reference Manual	Case study	Case analysis
	
		Respond with legitimating statements when a victim of ABE expresses shock (A)	Chapter 3 in HIV Reference Manual	Role play	Observation checklist
	
		Demonstrate capacity to apply universal precautions and waste management (S)	Chapter 3 in HIV Reference Manual	Clinic rotation	Observation checklist

(K) = Knowledge, (S) Skill, (A) Attitude

As noted, a key tenet of competency-based education is moving away from rote memorization or knowledge acquisition to the application of knowledge and skills. As such, the 'Teaching Guide' places great emphasis on a mix of interactive teaching methods to stimulate active student participation, such as case-based learning, role plays, and group discussions. The 'Teaching Guide' ensures integration between theory and practice, as many of the course plans specify practice-based experience with nurse monitors in a clinic setting.

Typically, the sole form of assessment in Haiti nursing schools is a final written examination, of essay, short answer or multiple-choice type. However, such examinations tend to reward rote recall of facts and don't assess a student's ability to apply knowledge in practice. The 'Teaching Guide' emphasizes structured observation as an alternative assessment method and emphasizes periodic assessment at regular intervals throughout each course. The final exam for nurses to obtain their license to practice will also be modified to reflect the new competencies.

It has been noted that faculty development is probably the single most necessary precursor to the successful implementation and maintenance of curricular reform [18,19]. Unless faculty members embrace the new content, expand their own knowledge base, and successfully integrate the new content into the curricula, curriculum reform simply cannot be made. To that end, a series of faculty development workshops have recently begun on the new content of the HIV/AIDS curriculum and on how to lead interactive teaching methodologies that not only enhance student knowledge but skills and attitudes. The curriculum committee will be working with faculty from each school in the coming months to design checklists that enable observation and judgments to be made about the students' mastery of the learning objectives. Curriculum committee members are also performing periodic site visits to the nursing schools to observe teaching activities, mentor faculty, and monitor and evaluate the implementation of the curriculum package.

Over the next four years, as students progress from Year 1 through Year 4 of the degree program, the HIV/AIDS curriculum will be evaluated formally in all four schools. In addition, data on faculty use of and satisfaction with the curriculum will be collected through semi-structured qualitative interviews and observation, the results of which will be used to identify any weaknesses and needed changes to the Teaching Guide or Reference Manual, as related to the level of difficulty, time allocation, content updates, or other areas. A revision schedule has not been set for the teaching material, as another goal of faculty development will be to build their skills in maintaining their currency in their field and to reflect this in lesson planning.

## Discussion

The effect of this change has broad implications for the Haiti nursing education community. All nursing students will now need to demonstrate mastery of HIV/AIDS-related competencies during periodic assessment with direct observation of the learner performing authentic tasks. Using what they learned in the faculty development workshops and the instructions and model exercises in the 'HIV/AIDS Teaching Guide', faculty will have the added responsibility of developing exercises to address the required competencies and creating assessment tools to demonstrate that their graduates have met the competencies. The major challenges in the next step will be creating assessment tools that are reliable, valid, and practical in this developing country setting.

There were several lessons learned from the process of developing the HIV competencies and integrating them into an already established broader nursing curriculum. The first lesson was the importance in identifying the right stakeholders for both the coordinating committee and the curriculum working groups. For both groups, bringing a multidisciplinary group of officials, faculty, administrators and HIV experts enriched the process, garnered buy-in, and improved the outcome by virtue of the collaborative process. The curriculum working group was made up of dedicated nurse leaders who were passionate about elevating the profile of nursing education in Haiti and graduating students competent to care for the large number of people living with HIV and AIDS.

The second lesson was that this activity brought different nursing schools together to collaborate on a shared goal that was manageable and timely using a process that could be repeated for other aspects of curriculum reform. Haiti's nursing schools face numerous challenges: lack of funds, lack of available clinical mentors, poor infrastructure, lack of curriculum developers, etc. Against this backdrop, other aspects of the overall nursing curriculum program need major reform but addressing only one topic through this systematic process gave the schools a manageable victory. It is hoped that this provided stakeholders with the experience, skills and motivation to strengthen other domains of the pre-service nursing curriculum, improve the synchronization of didactic and practical training, and develop standardized competency-based examinations for nursing licensure in Haiti. Each of these goals is part of the Ministry of Health's strategic plan for 2005–10 [20].

The third lesson was that defining competencies and related learning objectives, though absolutely essential to clarifying what students must learn, was conceptually difficult for the curriculum committee. Even experienced educators may find it challenging to clearly state the knowledge, skills, and attitudes underpinning a competency. Writing clear and measurable learning objectives, particularly attitudinal objectives, was challenging for the committee, and required a great deal of debate and revision.

It is necessary to develop the evidence base on the impact of pre-service curriculum strengthening initiatives in developing countries like the one described here [21]. There is not one HIV care delivery model in Haiti, meaning that pre-service programs have to provide flexible education which will allow nurses to integrate into settings with varied types of HIV-related services and with varied staffing patterns. Applied research is needed in settings like Haiti on the optimal role of nurses in support of HIV scale-up, the integration of HIV care and treatment with other components of primary care services, and the relationship between pre-service nursing training, quality of care, and patient health outcomes. On-going evaluation and documentation of Haiti's pre-service training initiative for nurses will hopefully yield insights useful for other settings and professional disciplines.

## Conclusion

In light of the critical role that nurses play in the care of Haiti's population, investing in pre-service nursing education institutions to improve the quality of HIV/AIDS training is a critical part of increasing the overall quality of HIV/AIDS care and treatment in the country. Education in HIV/AIDS is now an integral part of the four national nursing schools in Haiti. This was achieved using a multi-disciplinary, participatory process that can be applied to future curriculum reform efforts.

## Competing interests

The authors declare that they have no competing interests.

## Authors' contributions

EK provided technical assistance to the nursing committee and drafted the manuscript. NP conceived of the intervention, participated in its coordination, and helped to draft the manuscript. AD supported the technical committee. RD and MP participated in the design and implementation of the intervention. All authors read and approved the final manuscript.
